# The effects of intrapartum synthetic oxytocin on maternal postpartum mood: findings from a prospective observational study

**DOI:** 10.1007/s00737-018-0913-3

**Published:** 2018-10-10

**Authors:** Lea Takács, Jitka Mlíková Seidlerová, Zuzana Štěrbová, Pavel Čepický, Jan Havlíček

**Affiliations:** 1 Department of Psychology, Faculty of Arts, Charles University, Celetná 20, 116 42 Prague, Czech Republic; 2 Internal Department II, Faculty of Medicine in Pilsen, Charles University, Prague, Czech Republic; 3 Biomedical Centre, Faculty of Medicine in Pilsen, Charles University, Prague, Czech Republic; 4 Department of Zoology, Faculty of Science, Charles University, Viničná 7, 128 43 Prague, Czech Republic; 5 National Institute of Mental Health, Topolová 748, 250 67 Klecany, Czech Republic; 6 Department of Obstetrics and Gynecology, Na Bulovce Hospital, The First Faculty of Medicine, Charles University, Budínova 67/2, 180 00 Prague, Czech Republic

**Keywords:** Depression, Pregnancy and postpartum, Synthetic oxytocin, Maternity blues

## Abstract

Postpartum depression (PPD) affects up to 19% of all mothers, with detrimental effects on both mother and child. The antidepressant and anxiolytic effects of plasma oxytocin are well-documented, but it is still disputable whether synthetic oxytocin (synOT) may protect women against postpartum mood alterations. The current study examined the association between synOT intrapartum and maternal mood postpartum using a prospective design. Two hundred sixty women were screened for depressive symptoms in the last trimester of pregnancy and then again 6 weeks and 9 months postpartum using the Edinburgh Postnatal Depression Scale. They also completed Maternity Blues Questionnaire in the first postpartum week. The data concerning the intrapartum interventions and health status of the newborn were extracted from the medical records. Cox proportional hazards regression adjusted for a history of depression, mode of delivery, and childbirth experience showed that synOT predicted a significantly lower risk of PPD (HR = 0.65, 95% CI 0.45–0.95, *p* = 0.025). The risk factors for PPD included a history of depression (HR = 3.20, 95% CI 2.33–4.40, *p* < 0.001) and negative childbirth experience (HR = 1.39, 95% CI 1.01–1.90, *p* = 0.040). Logistic regression adjusted for the same covariates found no significant effect of synOT on maternity blues (OR = 0.64, 95% CI 0.31–1.32, *p* = 0.23). While synOT administered intrapartum does not affect maternal mood immediately, it may come to effect some weeks after childbirth to protect mothers from developing PPD symptoms.

**Electronic supplementary material:**

The online version of this article (10.1007/s00737-018-0913-3) contains supplementary material, which is available to authorized users.

## Introduction

Postpartum depression (PPD) affects up to 19% of all mothers (Gavin et al. 2005; Grace et al. 2003), with detrimental effects on both mother and child. PPD may alter maternal sensitivity and parenting competences (Murray et al. 1996) as well as mother-child relationship (Ohoka et al. 2014) and child development. Various studies found that children of the mothers affected are at an increased risk of delayed cognitive development (Cornish et al. 2005; Grace et al. 2003; Sutter-Dallay et al. 2011), impaired social development (Feldman et al. 2009; Matthey et al. 2005), including attachment insecurity (Forman et al. 2007), and behavioral difficulties (Grace et al. 2003).

Several studies observed inverse relationship between the plasma oxytocin (OT) levels in pregnant women and severity of prepartum or postpartum depressive symptoms (Garfield et al. 2015; Massey et al. 2016; Skrundz et al. 2011). Such findings are not surprising, given that OT is known to have antidepressant and anxiolytic effects (Heinrichs et al. 2003; Kirsch et al. 2005). Some authors point out however that it is rather the descending trajectory of OT levels during perinatal period than absolute OT levels as such what should be linked to PPD. Jobst et al. (2016), for example, found that in a non-PPD group, the OT plasma levels were continuously increasing between pregnancy and the postpartum period, while in a PPD group they were decreasing from the 38th week of gestation to 2 days after delivery.

Given the antidepressant and anxiolytic effects of OT, well-documented in previous studies, it may be hypothesized that administration of synthetic oxytocin (synOT) during the peripartum period ought to reduce the risk of a later PPD. However, a recent study (Kroll-Desrosiers et al. 2017) observed an opposite pattern, concluding that women who had been exposed to synOT intrapartum were more likely to experience depressive symptoms during the postpartum period. Similar findings were reported by Gu et al. (2016). Nevertheless, both studies have certain limitations that might weaken their findings. Having applied a retrospective design using data from a clinical data repository, Kroll-Desrosiers et al. (2017) identified exclusively the women with diagnosed and treated depression, while Gu et al. (2016), though having adopted a longitudinal design, did not take into account data regarding prior depressive symtpoms, which made impossible to distinguish between women at a high and a low risk of PPD.

The effect of intrapartum synOT exposure on maternal mood is of great concern given the relatively widespread administration of synOT to induce or augment labor and to prevent postpartum hemorrhage. In order to avoid the shortcomings of the previous studies, we investigated both short- and long-term effects of intrapartum synOT on maternal mood using a prospective longitudinal design and accounting for maternal history of depression and depressive symptoms in pregnancy. As previous studies reported a positive effect of plasma OT on mood and the aforementioned studies about the effects of intrapartum synOT on mood are limited, we hypothesized that women who had been exposed to synOT intrapartum would be at a lower risk of a mild early postpartum mood disturbance termed maternity blues (Watanabe et al. 2008) and subsequent PPD than women without such exposure and that the effects of synOT would be more apparent soon after delivery and attenuate over time.

## Materials and methods

### Participants and procedure

The present study is a part of a larger longitudinal research “The Mother and Child Vysočina Cohort Study” that examines perinatal determinants of family functioning and child development. This project was approved by the Ethics Committee of the Jihlava Hospital, which has ethical authorization powers in relation to the Vysočina Region where the study was carried out. Participants were recruited at five maternity hospitals in the Vysočina Region, Czech Republic (Havlíčkův Brod, Jihlava, Třebíč, Pelhřimov, Nové Město na Moravě) between October 2013 and September 2014 and followed regularly since then. All participants signed an informed consent form.

For this study, the data from the first four waves were analyzed: the last trimester of pregnancy (T1), 1 to 7 days after delivery (T2), 6 weeks postpartum (T3), and 9 months postpartum (T4). At T1, the screening inventory for depression (Edinburgh Postnatal Depression Scale, EPDS) (Cox and Holden 2003) was administered. As many women experience a mild transitory mood alteration termed postpartum blues, which occurs in the first days after delivery and lasts until 2 weeks postpartum, it has been recommended to screen for PPD not earlier than 2 weeks postpartum. The EPDS showed substantially higher sensitivity when administered later than 2 weeks after delivery compared to earlier period (Boyd et al. 2005). Thus, at T2, the Maternity Blues Questionnaire (MBQ) (Kennerley and Gath 1989) was administered along with a questionnaire concerning women’s sociodemographic background, anamnestic data, and childbirth experience. The EPDS was administrated at T3 and T4 again. The data concerning the course of labor, intrapartum interventions, and health status of the newborn were extracted from the medical records.

**Fig. 1:**
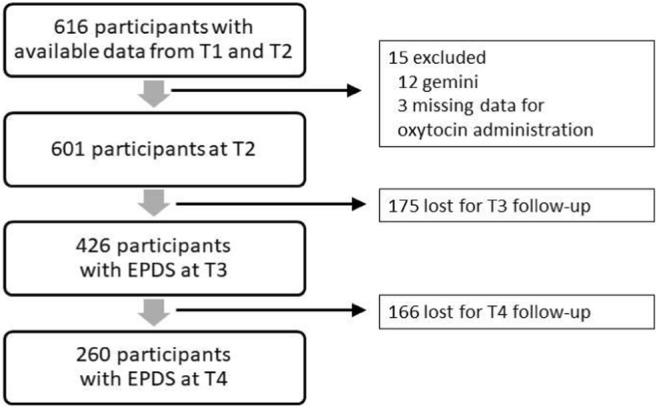
Data collected throughout the four stages of the study, T1 - third trimester of pregnancy; T2 - 1 to 7 days postpartum; T3 - 6 weeks postpartum; T4 - 9 months postpartum

A total of 966 women completed the questionnaire at T1. Among them, 847 women also completed the questionnaires at T2. We excluded respondents for whom data from medical records were not available (*n* = 231). We also excluded women for whom data about synOT administration were missing (*n* = 3) and those with multiple pregnancy (*n* = 12). After applying exclusion criteria, a total of 601 women had all data available for both T1 and T2. Of these, a number of 426 women completed the questionnaires at T3. Data from 260 women were available for all four waves of the data collection (T1–T4) (Fig. 1). The women who were lost to follow-up at T3 were less often married compared to women who completed the questionnaires at T1, T2, and T3 (60 vs. 73%; *p* = 0.003). No other differences were observed (see Supplementary material, Table A1). The same results were obtained when we compared women who were lost to follow-up at T3 to those who completed questionnaires at all four time points (see Supplementary material, Table A2).

### Measures

The participants reported depressive symptoms at T1, T3, and T4 on the Edinburgh Postnatal Depression Scale (EPDS) (Cox and Holden 2003). The EPDS is a 10-item self-report screening for PPD, validated also for the use with pregnant women (Breedlove and Fryzelka 2011). Each item was rated on a 4-point scale from 0 to 3. The total score may thus range from 0 to 30, with higher scores indicating higher levels of depression. The standard cutoff score > 12 (Cox and Holden 2003) was used in this study.

At T2, while still in maternity hospital, the participants completed the MBQ (Kennerley and Gath 1989). The MBQ consists of 28 items originally divided by means of cluster analysis into seven clusters (primary blues, reservation, hypersensitivity, decreased self-confidence, depression, despondency, and retardation). We applied a dichotomous scale for each symptom, where the women reported whether the given feeling was present or absent. The total score may thus range from 0 to 28. Positive screening for blues corresponded to the cutoff score values exceeding 90th percentile (Takacs et al. 2016).

At T2, women were asked to evaluate their childbirth experience on a scale from 1 to 7, with higher scores indicating less positive evaluation (a score above 5 was considered a cutoff for negative childbirth experience). They were also requested to mark how much stress they believed they experienced during childbirth on a 100-mm long visual analogue scale, with the anchoring phrases being “No stress at all.”/“The greatest stress I can imagine.” (a score above 75th percentile, i.e., > 68, was considered a stressful experience). Given the high correlation between those two items in our data, we created a composite variable “negative childbirth experience,” applying to women who scored above the cutoff point for at least one of the above questions.

### Statistical analysis

For statistical analysis, SAS software version 9.4 (SAS Institute Inc., USA) was used. The results are presented as arithmetic mean ± standard deviation, a proportion (percentage), or median (interquartile range). In order to assess the differences in sociodemographic characteristics between the groups (women with and without synOT exposure; women who dropped out the study and those who did not), we used Student *t* test, Fisher’s two-sided exact test or Wilcoxon two-sided test, respectively.

We searched for possible covariates of postpartum mood disturbance using Cox stepwise multiple regressions with the *p* values for independent variables to enter and to stay in the model set at 0.15. The dependent variable was postpartum mood disturbance at any time point of the study (T2, T3, or T4). The potential covariates included maternal age, parity, marital status, a history of depression (previous antidepressant treatment or score > 12 on EPDS in the last trimester of pregnancy), mode of delivery (spontaneous vaginal vs. operative delivery), women’s appraisal of childbirth experience, length of newborn’s postpartum stay in maternity hospital, and 10-min Apgar score.

We used Cox proportional hazards regression to examine the effect of synOT on postpartum mood disturbance (maternity blues/PPD). We regarded all mood assessments (blues at T2 and depressive symptoms at T3 and T4) as separate events and we considered all women to be at risk for mood disturbance at all measurement points, regardless of whether they scored above the cutoff on postpartum mood measures at any previous point of time. The marginal model was therefore chosen. In further analysis, we also added interaction terms between synOT and relevant factors (a history of depression, mode of delivery, and childbirth experience) to study potential moderating effects.

In addition, we conducted a sensitivity analysis where only data on PPD (i.e., from T3 and T4 assessments) were included in Cox proportional hazards regression, while maternity blues measured at T2 were not considered an event in this sub-analysis. To investigate the effect of synOT on postpartum mood in terms of time, we used logistic regression, where either maternity blues or postpartum depressive symptoms assessed at T4 were included as a dependent variable.

## Results

Out of 601 women who completed the questionnaires at both T1 and T2, 25.3% (*n* = 152) received synOT intrapartum. Compared to the non-synOT group, the women with synOT were younger and more frequently primiparous, with their children being hospitalized for longer periods postnatally (Table 1). No difference was found between the two groups regarding marital status, delivery mode, childbirth experience appraisal, severity of depressive symptoms in pregnancy, previous antidepressant treatment, or frequency of positive screening for PPD (cutoff > 12 on EPDS) 6 weeks postpartum. However, the women with synOT intrapartum scored less frequently above cutoff 12 on EPDS 9 months postpartum. There was a significant difference in duration of active phase of the first stage of labor (from cervical dilatation 3 cm until the complete dilatation) in primiparous women with and without synOT exposure to the effect that primarae who had received synOT had longer labors (Table 1).

**Table 1:** Characteristics of the sample (*n* = 601)

		SynOT intrapartum		*p*
		Yes (*n* = 152)	No (*n* = 449)	
Mean age ± SD		29.7 ± 4.0	30.5 ± 4.1	**0.036**
Primipara, *n* (%)		106 (69.7)	201 (44.8)	**< 0.001**
Marital status (married), *n* (%)		96 (63.2)	320 (71.3)	0.067
Mean newborn weight ± SD		3514 ± 523	3445 ± 464	0.14
10-min Apgar score		10 (10–10)	10 (10–10)	0.70
Postnatal hospitalization of the newborn, days, median, interquartile range		5 (4–6)	5 (4–5)	**0.023**
Negative childbirth experience, *n* (%)		54 (35.3)	133 (29.6)	0.19
Duration of active phase of 1st stage of labor in primiparae, minutes, median, interquartile range		227 (160–368)	182 (120–285)	**0.005**
Duration of active phase of 1st stage of labor in multiparae, minutes, median, interquartile range		161 (93–260)	141 (86–205)	0.086
Indication for synOT administration, *n* (%)	Induction of labor	38 (25)		
	Speeding up labor	79 (52)		
	Hemorrhage prevention	35 (23)		
Delivery type, *n* (%)	Spontaneous vaginal	102 (67.1)	302 (67.2)	
	Vaginal operative	15 (9.9)	7 (1.6)	
	Cesarean section	35 (23)	140 (31.2)	
	Planned CS	1 (0.7)	83 (18.5)	
	Emergency CS	34 (22.3)	57 (12.7)	
	Operative	50 (32.9)	147 (32.7)	1.00
Depression, *n* (%)	Previous antidepressant treatment or score > 12 on EPDS in pregnancy	17 (11.2)	56 (12.5)	0.77
	Previous antidepressant treatment	5 (3.3)	17 (3.8)	1.00
	EPDS > 12 in pregnancy	12 (7.9)	43 (9.6)	0.63
	EPDS > 12 six weeks postpartum (*n* = 426)	21 (20)	83 (25.9)	0.24
	EPDS > 12 nine months postpartum (*n* = 260)	5 (7.2)	35 (18.3)	**0.032**
Baby blues, *n* (%)	Baby blues score > 10 (> 90th percentile)	11 (7.2)	46 (10.2)	0.34

Values are means ± standard deviations in interval variables, frequencies (relative frequencies) in categorical variables and median (interquartile range) in ordinal variables. *p* for difference between the two categories were calculated using Student *t* test and Fisher’s two-sided exact test, or Wilcoxon two-sided test, respectively. Significant differences are marked in bold

In Cox stepwise multiple regressions evaluating the effect of potential covariates, only a history of depression, mode of delivery (operative vs. spontaneous vaginal), and women’s appraisal of childbirth experience were found to be significantly associated with postpartum mood disturbance. These covariates were therefore included in the Cox proportional hazards regression that was conducted to assess the effect of synOT on postpartum mood (postpartum blues/PPD). The results showed that the strongest risk factor for postpartum mood alteration was a history of depression (HR = 2.85, 95% CI 2.08–3.92, *p* < 0.001). The risk factors also included negative childbirth experience (HR = 1.57, 95% CI 1.17–2.11, *p* = 0.003) and operative delivery (HR = 1.46, 95% CI 1.09–1.96, *p* = 0.011). SynOT significantly predicted a lower risk of occurrence of postpartum mood disturbance (HR = 0.66, 95% CI 0.47–0.92, *p* = 0.014) (Table 2). The pattern of the results remained similar in the sensitivity analysis where other potentially confounding factors (maternal age, parity, marital status, and length of postnatal hospitalization of the newborn/10-min Apgar score) were included in the model (see Supplementary material, Table A3).

**Table 2:** Determinants of postpartum mood alteration (maternity blues/postpartum depression) - results of Cox proportional hazard regression (*n* = 601)

	HR (95% CI)	*p*
History of depression (previous antidepressant treatment or score > 12 on EPDS in pregnancy)	2.85 (2.08–3.92)	< 0.001
SynOT intrapartum	0.66 (0.47–0.92)	0.014
Operative delivery	1.46 (1.09–1.96)	0.011
Negative childbirth experience	1.57 (1.17–2.11)	0.003

We further explored whether synOT might interact with a history of depression, mode of delivery, and childbirth experience to affect postpartum mood. Cox proportional hazards regression identified no significant interaction between synOT and a history of depression or between synOT and mode of delivery. The interaction term for synOT and childbirth experience was marginally significant (*p* = 0.079). In women reporting positive childbirth experience, the protective effect of synOT was significant (HR = 0.47, 95% CI 0.27–0.80, *p* = 0.006), while there was no significant effect of synOT on postpartum mood in women evaluating their childbirth experience as negative (HR = 0.87, 95% CI 0.56–1.35, *p* = 0.54).

As a next step, we analyzed the effects of synOT on PPD only, excluding postpartum blues as an event from the analysis. Consequently, the data from PPD screening at T3 and T4 were considered without taking into account the data on maternity blues from T2. Cox proportional hazards regression adjusted for a history of depression, mode of delivery and childbirth experience appraisal (Table 3) showed similar results to those presented in Table 2, where maternity blues were considered an event along with PPD. The risk factors for PPD included a history of depression (HR = 3.20, 95% CI 2.33–4.40, *p* < 0.001) and negative childbirth experience (HR = 1.39, 95% CI 1.01–1.90, *p* = 0.040), but operative delivery was not a significant predictor of PPD. SynOT was found to be a protective factor against developing PPD (HR = 0.65, 95% CI 0.45–0.95, *p* = 0.025). Those results remained unchanged after adjustment for maternal age, parity, marital status, and length of postnatal hospitalization of the newborn/10-min Apgar score (see Supplementary material, Table A4).

**Table 3:** Determinants of postpartum depression - results of Cox proportional hazard regression (*n* = 426)

	HR (95% CI)	*p*
History of depression (previous antidepressant treatment or score > 12 on EPDS in pregnancy)	3.20 (2.33–4.40)	< 0.001
SynOT intrapartum	0.65 (0.45–0.95)	0.025
Operative delivery	1.21 (0.88 1.67)	0.24
Negative childbirth experience	1.39 (1.01–1.90)	0.040

Only women who completed the T3 questionnaires were included in this analysis

In order to disentangle the effects of synOT on blues and PPD, we conducted a logistic regression analysis where only maternity blues (assessed at T2) and not PPD were considered a dependent variable. In the analysis adjusted for a history of depression, mode of delivery and childbirth experience appraisal, we found no significant association between synOT and maternity blues (OR = 0.64, 95% CI 0.31–1.32, *p* = 0.23). None of the interaction terms (between synOT and a history of depression; operative labor; childbirth experience) was significant (*p* ≥ 0.24). Logistic regression adjusted for a history of depression, mode of delivery and childbirth experience appraisal, where only PPD measured at T4 was considered a dependent variable, showed a significant protective effect of synOT (OR = 0.34, 95% CI 0.12–0.95, *p* = 0.041).

## Discussion and conclusions

In line with our hypothesis, we observed that intrapartum exposure to synOT may decrease the risk of developing PPD symptoms. There was, however, no protective effect against postpartum blues. It therefore seems that while synOT administered intrapartum does not affect maternal mood immediately, it may come to effect some weeks after childbirth. This is surprising since we assumed that the effect of synOTwould be more intense sooner after its application.

The results of the present study contradict those obtained by Kroll-Desrosiers et al. (2017) and Gu et al. (2016), who found that women with intrapartum exposure to synOT were at a higher risk of subsequent depressive disorder than women without such exposure. This discrepancy could be explained by a non-uniform synOT usage. In fact, when used suboptimally, synOT may lead to an excessively short and painful labor and delivery or, on the contrary, to a longer labor with complications and interventions, which may in turn result in a negative or even traumatic birth experience that is associated with PPD (Lemola et al. 2007). Accordingly, the relationship between synOT administration and later depressive symptoms could be moderated by the subjective birth experience. Our data appear to support such assumption showing that women who evaluated their childbirth experience positively were at a lower risk of postpartum mood alteration provided that they received synOT, which was not true for women who rated their childbirth experience as negative. Those results should, however, be interpreted with caution, because the interaction term for the effects of synOT and childbirth experience appraisal was only marginally significant.

Since Kroll-Desrosiers et al. (2017) did not report any indicators of optimal/suboptimal synOT usage or childbirth experience appraisal, it cannot be ruled out that their sample included a considerable proportion of women whose birth experience was negative due to a suboptimal synOT administration. Gu et al. (2016) screened for symptoms of posttraumatic stress following childbirth and found them unrelated to synOT administration, but women with a negative childbirth experience do not necessarily develop posttraumatic stress symptoms. In our sample, primiparae with synOT experienced longer labors than primiparae without synOT. As the most frequent indication for synOT usage was failure to progress, we may assume that synOT was underdosed rather than overdosed in those cases, without exacerbating the childbirth pain and making the childbirth experience negative. As the appropriate synOT administration might be decisive in terms of subsequent effects on PPD, future studies should specifically address this issue.

The main strength of this study lies in its longitudinal prospective design and repeated measurements of maternal depressive symptoms, including screening for depression in pregnancy. Given the widely recognized fact that PPD is underdiagnosed and undertreated (Werner et al. 2015), it is an advantage of the present study that a screening for depression (the EPDS) was applied rather than a diagnosis of depression. It was then possible to detect women suffering from depressive symptoms with a higher sensitivity than in the study by Kroll-Desrosiers et al. (2017), where exclusively the cases of diagnosed or treated depression were considered. A potential limitation of our study can be seen in the fact that the timing and exact dosage of synOT administration could not be taken into account. However, a study by Hinshaw et al. (2008) showed that the timing of OT administration did not affect the occurrence of later depressive symptoms. Another limitation is that we did not control for participants’ endogenous OT levels. Doing so would be advisable because it is possible that women with lower endogenous OT levels are at a higher risk of both depression and insufficient uterine activity that requires synOT administration. Future studies should adopt a prospective design and assess postpartum depressive symptoms repeatedly at different points in time, including pre-partum period, while concurrently measuring the levels of endogenous OT. In this way, they could shed light on the mechanism of interaction between endogenous OT, synOT, and depression.

This study is the first to suggest that intrapartum synOT might be beneficial in PPD prevention. Nevertheless, our results must be viewed with caution as our study was observational in design and cannot therefore provide conclusions regarding causality. While controlling for the various potential covariates, we cannot rule out that the buffering effect of synOT on depressive symptoms was related to an unknown factor. Further research is needed to reinforce our findings before any clinical recommendations can be made.

## Electronic supplementary material

ESM 1 (DOCX 14.4 kb)

ESM 2 (DOCX 14.3 kb)

ESM 3 (DOCX 13.1 kb)

ESM 4 (DOCX 13.1 kb)
