# Supplementary material for: The effects of intrapartum synthetic oxytocin on maternal postpartum mood: findings from a prospective observational study
Source: Arch Womens Ment Health. 2018 Oct 10;22(4):485–91. doi: 10.1007/s00737-018-0913-3 (PMC6647378; doi:10.1007/s00737-018-0913-3)
Supplement: Supplementary file 1 — (DOCX 14.4 kb) [file 737_2018_913_MOESM1_ESM.docx]

**Supplementary material**

Table A1 Differences between women who dropped out at T3 and those who participated at T3

|  |  | Participation at T3 | |  |
| --- | --- | --- | --- | --- |
|  |  | Yes (n = 426) | No (n = 175) | p |
| Mean age±SD |  | 30.4±4.0 | 29.8±4.3 | 0.068 |
| Primipara, n (%) |  | 219 (51.4) | 88 (50.3) | 0.86 |
| Oxytocin administration, n (%) |  | 105 (24.6) | 47 (26.9) | 0.61 |
| Marital status (married), n (%) |  | 311 (73.0) | 105 (60.0) | **0.0025** |
| Mean newborn weight±SD |  | 3450±477 | 3493±488 | 0.32 |
| 10-minute Apgar score |  | 10 (10-10) | 10 (10-10) | 0.33 |
| Postnatal hospitalization of the newborn, days, median, interquartile range |  | 5 (4-5) | 5 (4-6) | 0.49 |
| Negative childbirth experience, n (%) |  | 136 (31.9) | 61 (29.1) | 0.56 |
| Indication for synOT administration, n (%) | Induction of labor | 32 (30.5) | 6 (12.8) |  |
|  | Speeding up labor | 51 (48.6) | 28 (59.6) |  |
|  | Haemorrhage prevention | 22 (20.9) | 13 (27.6) |  |
| Delivery type, n (%) | Spontaneous vaginal | 290 (68.1) | 114 (65.1) |  |
|  | Vaginal operative | 15 (3.5) | 7 (4.0) |  |
|  | Caesarean section | 121 (28.4) | 54 (30.9) |  |
|  | Planned CS | 60 (14.1) | 24 (13.7) |  |
|  | Emergency CS | 61 (14.3) | 30 (17.2) |  |
|  | Operative | 136 (31.9) | 61 (34.9) | 0.50 |
| Depression, n (%) | Previous antidepressant treatment  or score > 12 on EPDS in pregnancy | 45 (10.6) | 28 (16.0) | 0.074 |
|  | Previous antidepressant treatment | 14 (3.3) | 8 (4.6) | 0.48 |
|  | EPDS > 12 in pregnancy | 34 (8.0) | 21 (12.0) | 0.12 |
| Baby blues, n (%) | Baby blues score >10 (> 90^th^ percentile) | 34 (8.0) | 23 (13.1) | 0.065 |

Values are means±standard deviations in interval variables, frequencies (relative frequencies) in categorical variables and median (interquartile range) in ordinal variables. p for difference between the two categories were calculated using Student t-test, Fisher’s two-sided exact test or Wilcoxon two-sided test, respectively.
