# Supplementary material for: The effects of intrapartum synthetic oxytocin on maternal postpartum mood: findings from a prospective observational study
Source: Arch Womens Ment Health. 2018 Oct 10;22(4):485–91. doi: 10.1007/s00737-018-0913-3 (PMC6647378; doi:10.1007/s00737-018-0913-3)
Supplement: Supplementary file 2 — (DOCX 14.3 kb) [file 737_2018_913_MOESM2_ESM.docx]

**Supplementary material**

Table A2 Differences between women who dropped out at T3 and those who attended both T3 and T4

|  |  | Participation at both T3 and T4 | |  |
| --- | --- | --- | --- | --- |
|  |  | Yes (n=260) | No (n=175) | p |
| Mean age±SD |  | 30.5±3.8 | 29.8±4.3 | 0.074 |
| Primipara, n (%) |  | 137 (52.7) | 88 (50.3) | 0.63 |
| Oxytocin administration, n (%) |  | 69 (26.5) | 47 (26.9) | 1.00 |
| Marital status (married), n (%) |  | 182 (70.0) | 105 (60.0) | **0.039** |
| Mean newborn weight±SD |  | 3460±469 | 3493±488 | 0.49 |
| 10-minute Apgar score |  | 10 (10-10) | 10 (10-10) | 0.27 |
| Postnatal hospitalization of the newborn, days,  median, interquartile range |  | 5 (4-5) | 5 (4-6) | 0.35 |
| Negative childbirth experience, n (%) |  | 84 (32.3) | 61 (29.1) | 0.53 |
| Indication for synOT administration, n (%) | Induction of labor | 17 (24.6) | 6 (12.8) |  |
|  | Speeding up labor | 35 (50.8) | 28 (59.6) |  |
|  | Haemorrhage prevention | 17 (24.6) | 13 (27.6) |  |
| Delivery type, n (%) | Spontaneous vaginal | 172 (66.1) | 114 (65.1) |  |
|  | Vaginal operative | 11 (4.2) | 7 (4.0) |  |
|  | Caesarean section | 77 (29.7) | 54 (30.9) |  |
|  | Elective CS | 36 (13.9) | 24 (13.7) |  |
|  | Emergency CS | 41 (15.8) | 30 (17.2) |  |
|  | Operative | 88 (33.8) | 61 (34.9) | 0.84 |
| Depression, n (%) | Previous antidepressant treatment  or score > 12 on EPDS in pregnancy | 36 (13.8) | 28 (16.0) | 0.58 |
|  | Previous antidepressant treatment | 11 (4.2) | 8 (4.6) | 1.00 |
|  | EPDS > 12 in pregnancy | 28 (10.8) | 21 (12.0) | 0.76 |
| Baby blues, n (%) | Baby blues score >10 (> 90^th^ percentile) | 21 (8.1) | 23 (13.1) | 0.10 |

Values are means±standard deviations in interval variables, frequencies (relative frequencies) in categorical variables and median (interquartile range) in ordinal variables. p for difference between the two categories were calculated using Student t-test, Fisher’s two-sided exact test or Wilcoxon two-sided test, respectively.
