# Supplementary material for: The effects of intrapartum synthetic oxytocin on maternal postpartum mood: findings from a prospective observational study
Source: Arch Womens Ment Health. 2018 Oct 10;22(4):485–91. doi: 10.1007/s00737-018-0913-3 (PMC6647378; doi:10.1007/s00737-018-0913-3)
Supplement: Supplementary file 3 — (DOCX 13.1 kb) [file 737_2018_913_MOESM3_ESM.docx]

**Supplementary material**

Table A3 Determinants of postpartum mood alteration (maternity blues/postpartum depression) – results of Cox proportional hazard regression (n = 601)

|  | HR (95% CI) | p |
| --- | --- | --- |
| History of depression (previous antidepressant treatment  or score > 12 on EPDS in pregnancy) | 2.82 (2.05 – 3.89) | <0.0001 |
| Oxytocin administration | 0.65 (0.46 – 0.91) | 0.014 |
| Operative delivery | 1.44 (1.07 – 1.95) | 0.011 |
| Negative childbirth experience | 1.55 (1.15 – 2.08) | 0.0029 |
| Maternal age | 0.99 (0.96 – 1.03) | 0.75 |
| Primiparity | 1.02 (0.72 – 1.46) | 0.89 |
| Postnatal hospitalization of the newborn | 1.01 (0.96 – 1.07) | 0.59 |
| Marital status (married) | 1.07 (0.78 – 1.46) | 0.67 |

We performed this analysis including either length of postnatal hospitalization of the newborn or 10-minute Apgar score. Here we report the model where length of postnatal hospitalization of the newborn was included. The results for 10-minute Apgar score were similar.
