# Supplementary material for: The effects of intrapartum synthetic oxytocin on maternal postpartum mood: findings from a prospective observational study
Source: Arch Womens Ment Health. 2018 Oct 10;22(4):485–91. doi: 10.1007/s00737-018-0913-3 (PMC6647378; doi:10.1007/s00737-018-0913-3)
Supplement: Supplementary file 4 — (DOCX 13.1 kb) [file 737_2018_913_MOESM4_ESM.docx]

**Supplementary material**

Table A4 Determinants of postpartum depression – results of Cox proportional hazard regression (n = 426)

|  | HR (95% CI) | p |
| --- | --- | --- |
| History of depression (previous antidepressant treatment  or score > 12 on EPDS in pregnancy) | 3.04 (2.22 – 4.15) | <0.0001 |
| Oxytocin administration | 0.67 (0.46 – 0.98) | 0.038 |
| Operative delivery | 0.88 – 1.68 | 0.24 |
| Negative childbirth experience | 1.44 (1.05 – 1.97) | 0.024 |
| Maternal age | 0.98 (0.94 – 1.03) | 0.30 |
| Primiparity | 0.74 (0.51 – 1.08) | 0.11 |
| Postnatal hospitalization of the newborn | 1.01 (0.96 – 1.07) | 0.63 |
| Marital status (married) | 1.24 (0.89 – 1.73) | 0.20 |

Only women who completed the T3 questionnaires were included in this analysis. We performed this analysis including either length of postnatal hospitalization of the newborn or 10-minute Apgar score. Here we report the model where length of postnatal hospitalization of the newborn was included. The results for 10-minute Apgar score were similar.
